# Female Gonadal Venous Insufficiency in a Clinical Presentation Which Suggested an Acute Abdomen—A Case Report and Literature Review

**DOI:** 10.3390/medicina59050884

**Published:** 2023-05-04

**Authors:** Sergiu-Ciprian Matei, Cristina Ștefania Dumitru, Andrei-Ion Oprițoiu, Lucian Marian, Marius-Sorin Murariu, Sorin Olariu

**Affiliations:** 1 Abdominal Surgery and Phlebology Research Center, “Victor Babeș” University of Medicine and Pharmacy, Eftimie Murgu Sq. No. 2, 300041 Timișoara, Romania; matei.sergiu@umft.ro (S.-C.M.); murariu.marius@umft.ro (M.-S.M.); srnolariu@yahoo.com (S.O.); 2 1st Surgical Clinic, “Pius Brînzeu” Emergency County Hospital, Liviu Rebreanu Boulevard No. 156, 300723 Timișoara, Romania; andrei_opritoiu@yahoo.com; 3 Department of Microscopic Morphology/Histology, Angiogenesis Research Center, “Victor Babes” University of Medicine and Pharmacy, Sq. Eftimie Murgu No. 2, 300041 Timișoara, Romania; 4 Urology Clinic, “Pius Brînzeu” Emergency County Hospital, Liviu Rebreanu Boulevard No. 156, 300723 Timișoara, Romania; lucimarian93@gmail.com

**Keywords:** venous insufficiency, varicose veins, female varicocele, surgery, interventional radiology

## Abstract

Pelvic venous insufficiency (PVI) is frequently associated with symptoms of abdominal pain or discomfort that is overlooked or under-diagnosed in women. Despite the fact that pelvic venous insufficiency in men is very well documented, its occurrence in women needs to be further studied. Patients with pelvic varicose veins undergo a long and inconclusive diagnostic work-up before the exact cause of the symptoms is identified. Gonadal venous insufficiency (GVI) is a condition that can present acutely, leading to diagnostic challenges. We present a case report of a 47-year-old female with acute abdominal pain and GVI, where endovascular embolization was used for successful treatment. The patient was diagnosed with GVI based on imaging findings of an enlarged left ovarian vein with retrograde flow and dilated pelvic veins seen on magnetic resonance imaging (MRI) with contrast material. Due to the severity of her symptoms and imaging findings, endovascular embolization was chosen as the treatment modality. The embolization was successful, and the patient’s symptoms resolved completely. This case highlights the challenge of diagnosing GVI with acute clinical expression and the potential benefits of endovascular embolization as a treatment option. Further studies are needed to determine the optimal management strategies for acute GVI, but endovascular embolization should be considered a safe and effective option. At the same time, we present a short review of the recent literature data related to this topic.

## 1. Introduction

Varicocele, a varicosity of the gonadal venous plexus, is a well-recognized disorder, occurring in up to 10% of men. In women, an analogous varicosity of the salpingo-ovarian plexus is rare. This may be explained, in part, by the lack of clinical findings on objective examination in women compared with men and the need, until recently, to use invasive venographic methods to confirm the diagnosis [1]. In some cases, vulval varices and perivulval veins may be observed, which area consequence of the incompetence of one or both ovarian veins [2]. When female patients complain of chronic pelvic pain of unknown etiology, one should look for atypical varices and vulval varices and consider pelvicvein insufficiency as a possible cause [3].

Venous disorders of the pelvis are associated with a spectrum of symptoms arising from reflux, most commonly involving the gonadal and internal iliac veins, and obstruction, usually of the left renal and iliac veins. These hemodynamic patterns are associated with at least four broad clinical presentations, including left flank or abdominal pain and hematuria, chronic pelvic pain, venous claudication, and symptomatic lower extremity varicosities in either atypical or typical saphenous distributions, the latter frequently recurring after initial treatment. The relationship between pelvic symptoms and venous pathology is far more complex than in the lower extremity. Multiple symptoms may be present concurrently, and several potential pathophysiologic mechanisms, such as left renal and iliac venous compression, may be simultaneously present. Additionally, similar symptoms may arise from disparate underlying causes (chronic pelvic pain can arise from primary ovarian vein reflux, left common iliac vein compression, or left renal vein compression), and similar anatomic derangements may lead to different symptoms (left renal vein compression may be associated with either left flank pain and hematuria or chronic pelvic pain). This can lead to diagnostic errors and may be responsible for the suboptimal results of many interventions [4].

Transvaginal duplex ultrasound may be a feasible investigation, and may be useful in terms of diagnosis. The sonographic appearances of the ovarian and pelvic varicocele are multiple dilated veins around the ovary and uterus with a venous Doppler signal of varying amplitude. The presence of circular or linear venous structures with a diameter >5 mm is indicative of pelvic varicosities [5]. Other superior non-invasive imaging modalities, such as computed tomography or magnetic resonance imaging, and invasive catheter-based venography, can help characterize varicosities and venous insufficiency, supporting the diagnosis [6].

## 2. Materials and Methods

Detailed Case Description. A 47-year-old woman presented herself to the emergency department complaining of diffuse lower abdomen pain, the slowing of intestinal transit, and fatigue. The painful symptomatology affirmatively started about two weeks prior with mild intermittent episodes, which had worsened in the last few hours. The local clinical examination revealed an abdomen with normal adipose panniculus (body mass index = 24.17 kg/m^2^), mobile with respiratory movements, spontaneously painful in the lower site, with tenderness and slight muscular defense during palpation, especially in the left iliac region. From the patient’s medical history, we noted 3 pregnancies with 3 natural births and high blood pressure treated with Perindopril 5 mg/Indapamide 1.25 mg, one tablet/day. Blood tests did not reveal significant pathological modifications. The patient was admitted to the Surgical Department for further investigation and treatment.

In order to exclude a urological condition, we performed ultrasonography of the kidneys, ureters, and bladder (USG-KUB), and it indicated that both kidneys are of normal size, shape, and echo pattern with normal cortical thickness. No calculus, growth, or hydronephrotic changes were seen in either kidney. Corticomedullary junctions of both kidneys were sharply defined. The bladder was moderately distended and did not show any intraluminal pathology. Upon the physical examination of the patient, the bladder dropped only a short way into the vagina, so a mild cystocele (type 1) was seen. The microscopic exam of the urine was normal. A uroflowmetry was performed with a voided urine volume of 280 mL and a maximum flow rate of 16 mL/s; therefore, it excluded a urodynamic pathology. Post-voiding residual volume showed that the bladder was emptied completely.

**Figure 1:**
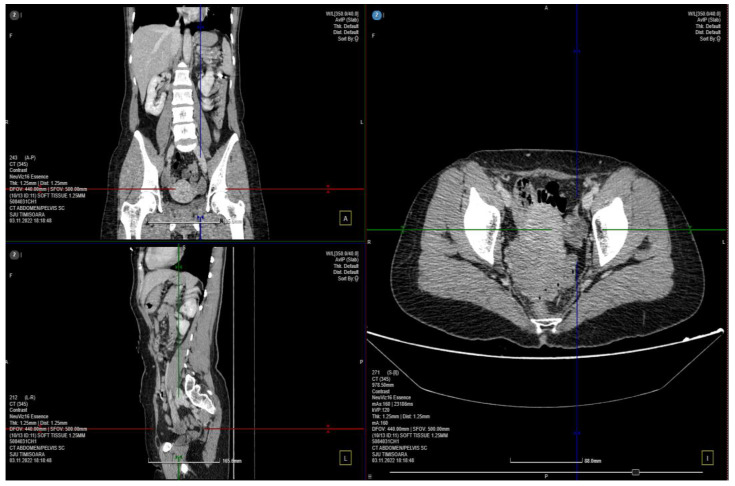
CT scan of the abdomen and pelvic 3 different plans: the coronal, sagittal and axial planes.

A computed tomography (CT) scan was performed, and a few images of the cystic characters on the left ovary were observed without signs of local complication (Figure 1). The parauterine vasculature was up to 5 mm in diameter, and the caliber is reserved for the examination of the patient in the supine position. No specific signs of an acute inflammatory pathology, such as appendicitis or diverticulitis, were noted. Uterus and adnexa are difficult to assess accurately through the use of current investigation–recommended correlation with gynecological (GYN) examination and completion using pelvic magnetic resonance imaging (MRI) with a contrast material.

**Figure 2:**
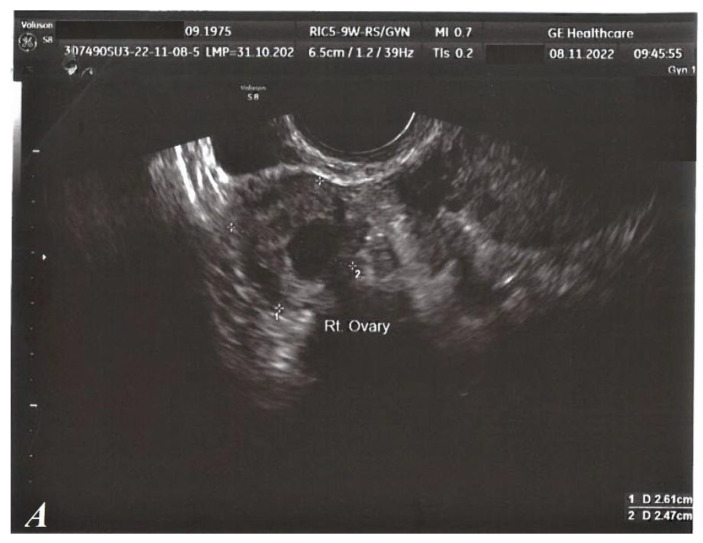

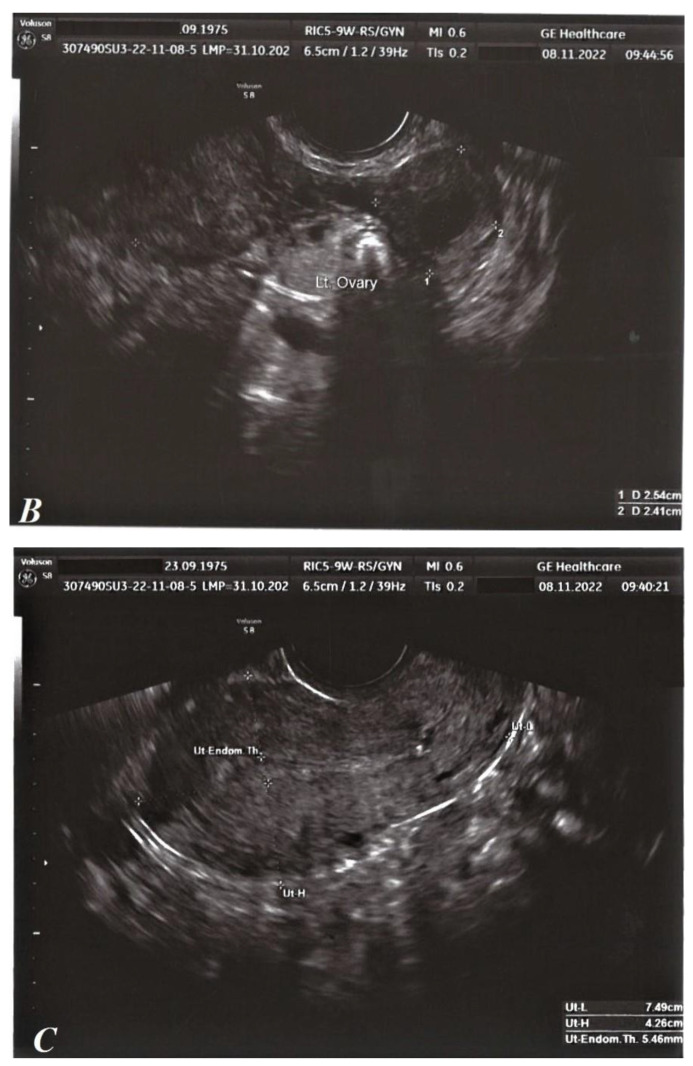
TVUS, normal aspect. Right ovary (**A**). Left ovary (**B**). Uterus (**C**).

Regarding local GYN examination, as well as standard transvaginal ultrasound (TVUS), no clinical pathological signs were detected. (Figure 2).

**Figure 3:**
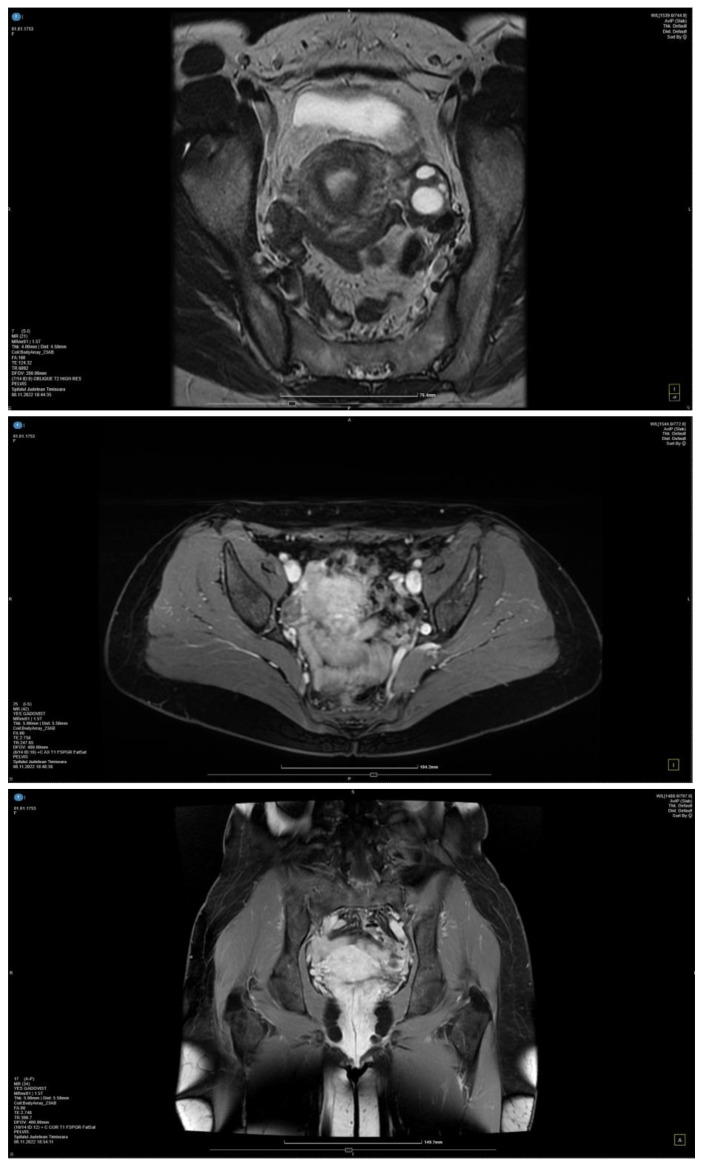
MRI with contrast material in 2 different planes: axial (I) and coronal (A) planes.

The MRI exam using contrast material revealed a thin blade of free intrapelvic fluid (infracentimetric) at the level of the posterior recess, which, in a physiological context, is as follows: a few bilateral, millimetric, functional-looking ovarian cysts ≤5 mm (follicular cysts); parauterine vasculature with dilated (about 7 mm), tortuous, arcuate, T2 hyperintense and T1 flow voids appearance; and the appearance of simple Naboth cyst with dimensions of 4/2 mm (Figure 3). The diagnosis of pelvic venous congestion syndrome due to left ovarian vein insufficiency was established.

**Figure 4:**
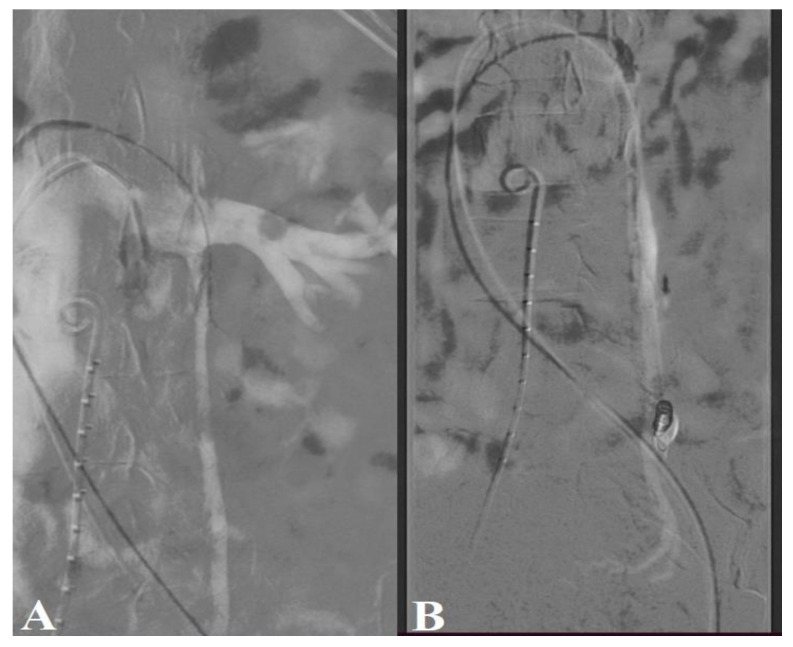
CT-guided endovascular embolization. The left ovarian vein was catheterized via transfemoral approach, respectively, through the left renal vein (**A**). In venograms performed during the Valsalva maneuver, left ovarian vein insufficiency with paraovarian varices was detected (**B**).

CT-guided endovascular embolization of the left ovarian vein was decided as the chosen therapeutic method. The puncture of the left common femoral vein was performed with the installation of a 5F access sheath. The left renal vein was subsequently catheterized with a Cobra 2 5F catheter and a 0.035″ hydrophilic guide. When injecting into the left renal vein simultaneously with the Valsalva maneuver, reflux was evident in the left ovarian vein, with this having an increased caliber of approximately 1.1 cm. The left ovarian vein was selectively catheterized with a 0.035″ hydrophilic 260 cm guide, later changing the catheter to a vertebral glide 4F, and advanced to the middle third portion of this vein. The control injection showed congested left parauterine veins. The distal portion of the ovarian vein was super-selectively catheterized with a 0.021″ microcatheter, followed by the placement of a 6/20 mm detachable coil (Interlock, Boston Scientific, MA, USA). On the microcatheter placed in the distal portion of the ovarian vein, a mixture of lipiodol and acrylic glue (GLUBRAN^®^, GEM, Milan, Italy), ratio 1:1, was injected, with the synchronous withdrawal of the microcatheter until the vein was closed (Figure 4). During the control injection in the left renal vein, reflux in the ovarian vein was no longer objectified.

Patient evolution was favorable, and she was discharged 24 h after the intervention, with phlebotonic medications with diosmin600 mg, one tablet per day, being recommended. The patient was evaluated clinically and paraclinically (blood tests and abdominal and pelvic ultrasound at 7 days and one month). At six months, a control MRI was additionally performed, with no complications being encountered. In order to evaluate the superficial venous network of the lower limbs, a duplex ultrasound was performed. No signs of venous reflux were identified.

## 3. Discussion

When pain appears due to pelvic vein insufficiency, it is most often chronic, and it can be associated with other non-specific manifestations, including dysmenorrhea, dysuria, nausea, bloating, and abdominal and rectal pain [7]. Precisely due to the lack of clinical signs, in the case of female patients who complain of chronic pelvic pain of unknown etiology, pelvic vein insufficiency should be considered to be a possible cause [3]. The particularity of the case we presented is the relatively acute onset of abdominal pain, which raised suspicion of an acute abdominal inflammatory pathology, associated with the apparent lack of clinical and anamnestic data, which subsequently raised suspicion of gonadal venous insufficiency. However, following the initial paraclinical investigation, an acute abdominal inflammatory pathology was ruled out, and a diagnosis was relatively difficult to establish. Due to the absence of clinical signs, the diagnosis was not clarified even after the gynecological clinical examination, with MRI being needed. The combination of postcoital pain and ovarian tenderness upon clinical examination is very suggestive in terms of the diagnosis [8], but those signs were not encountered in our case. A transvaginal Doppler ultrasound during the gynecological consultation would have been useful instead and would have identified the cause of the pain. To diagnose this condition, various invasive and non-invasive imagistic procedures are available. Non-invasive modalities, including ultrasonography (US), magnetic resonance imaging (MRI), and computed tomography (CT), can also exclude other pathologies; however, their sensitivity in the diagnosis of pelvic venous insufficiency (PVI) remains low [9]. Therefore, venography remains the modality of choice. However, interpretations vary and catheterizing pathological afferents is difficult, particularly at the pelvic level [10,11]. Venography is also invasive and generally precedes therapeutic interventions. Furthermore, chronic pelvic pain often leads to gynecologic diagnostic laparoscopies. According to certain reports, about 40 percent of those laparoscopic procedures are related to chronic pelvic pain. The rate of the occurrence of pathological findings identified through the use of laparoscopies in women with chronic pelvic pain range between 35 and 83%. In about twenty percent of these cases, pelvic congestion is also identified [12].

Due to the fact that our patient was hospitalized in the surgery department with the suspicion of an acute abdominal pathology, even if this was later excluded through the use of an MRI examination that established diagnostic certainty, it is necessary to follow several elements of differential diagnosis, which includes acute surgical diseases encountered in the lower abdomen, such as appendicitis, Meckel diverticulum, bowel obstruction, or ovary torsion, as well as the differential diagnosis of pelvic congestion syndrome. The list of differential diagnoses for pelvic congestion syndrome is vast, including diseases of the urinary tract, gastrointestinal tract, musculoskeletal disorders, disorders of neurological origin, gynecological problems, and mental health disorders. Painful bladder syndrome, pelvic inflammatory disease, interstitial cystitis, endometriosis, pelvic neuralgia, irritable bowel syndrome, myofascial pain, and pelvic floor myalgia are the common causes of chronic pelvic pain. In some cases, formulating an accurate diagnosis of the underlying causes of chronic pelvic pain is difficult, even with the use of laparoscopic and diagnostic radiological tests [12,13,14]. Due to the non-specific symptoms and the difficulty of establishing a diagnosis in our case, a urological differential diagnosis was necessary with the aim of ruling out life-threatening pathologies. The relevant conditions to be considered in this case are the spontaneous rupture of the distal ureter and bladder rupture. The spontaneous rupture of the distal ureter is a rare condition with no plausible explanation; only theoretical mechanisms have been proposed. Impacted calculi on the ureteral wall or a stone moving downward can erode and ulcerate the ureteral wall, leading to ureteral rupture at the distal ureteral obstruction. Additionally, malignancy, idiopathic retroperitoneal fibrosis, bladder outlet obstruction, and some connective tissue diseases that cause fibrotic changes have also been proposed as causes of spontaneous ureteral rupture. Spontaneous ureteral rupture has no characteristic clinical signs. Upon physical examination, patients may present with abdominal tenderness and pain, and, in some cases, diagnosis may be difficult due to the non-specific symptoms [15]. In the current case, no evidence of any underlying pathological condition was found. Bladder rupture is a rare complication related to bladder cancer with a high mortality rate. Since bladder rupture is an emergency, the diagnosis and treatment of the cancer are usually delayed. Spontaneous bladder rupture usually presents with acute abdominal pain and may go undiagnosed for a period ranging from days to weeks, and clinical suspicion is key to early diagnosis [16]. In the presented case, a CT scan showed no evidence of intraluminal bladder mass or peri vesical build-up fluid; therefore, perforation was excluded.

Generally, this condition is mainly caused by pregnancy, and in rare cases, ovarian and internal iliac venous insufficiency occurs due to valvular incompetence or the compression of the left renal or left common iliac vein. The hormonal impregnation of estrogens and progesterone causes significant dilation, a loss of elasticity in the venous wall, and the separation of the valve walls, which are responsible for venous reflux and a failure to return to normal in the months following childbirth [16]. The etiology of this condition, in our case, is most probably represented by previous pregnancies. This hypothesis, related to women who have had multiple pregnancies, is the most accepted in the literature [8].

Several options have proven to be effective in pain resolution, including medical treatment, surgery, and percutaneous embolization, which have become the standard treatment for pelvic venous insufficiency and results in symptomatic improvements in 80–94% of patients [17,18,19,20]. It seems that endovascular treatments applied with just the use of a coil had a significantly greater outcome in pain management than those that involved the use of various additional materials in combination. Additionally, it was observed that patients with unilateral ovarian venous insufficiency before the procedure were more successful in terms of pain management than those with bilateral insufficiency [21].

With the development of medical technologies, many treatment methods for pelvic venous insufficiency have been developed. Currently, the treatment of pelvic engorgement syndrome and pelvic varicose veins with coil embolization, plugs, or transcatheter sclerotherapy, used alone or together, are recommended by the Society for Vascular Surgery (SVS)/American Venous Forum (AVF) guidelines (GRAD 2B) [4,22]. The first endovascular approach for PVI was introduced by Edwards et al. in 1993. Endovascular treatment is a currently widely used and well-tolerated procedure with higher efficacy, fewer risks, and an easier recovery than surgical treatment [23]. Initially, this treatment was used as an alternative to surgical ligation of the ovarian veins and later became a gold standard [11,24]. In the case of bilateral female gonadal venous insufficiency, the most controversial issue is the choice of vessels to be embolized. There are different opinions in the literature, some suggesting that only the embolization of the left ovarian vein may be sufficient [22], while other authors argue that both ovarian veins should be closed [25].

On the other hand, other therapeutic alternatives are currently available for patients suffering from severe dilatation and reflux in the gonadal veins, such as the use of progesterone [26,27]. It causes some degree of venoconstriction within the ovarian vessels, which can relieve some of the symptoms in the short term. Other drugs that have been used in the treatment of PVI are vasoactive agents. These drugs are designed to improve venous tone and reduce venous distension. One of the most extensively studied vasoactive agents is diosmin, a flavonoid extracted from citrus fruits. A randomized, double-blind, placebo-controlled trial found that diosmin was effective in reducing pain and improving the quality of life in women with chronic pelvic pain attributed to PVI [28]. Another randomized controlled trial found that a combination of diosmin and hesperidin, another flavonoid, was effective in reducing pelvic pain and improving the quality of life in women with PVI [29]. However, recent studies recommend 600 mg of pure diosmin as the best phlebotonic medication option in terms of patient compliance, symptom improvement, and the long-term prevention of recurrences [30,31]. It is important to note that drug treatments for PVI are not a cure and are primarily aimed at managing the symptoms of the condition. Lifestyle modifications, such as regular exercise and weight loss, may also be effective in reducing the symptoms of PVI. In severe cases, surgical interventions, such as embolization or the stenting of the affected veins, may be necessary [32,33].

PVI, although a clinical entity, is not properly evaluated and, in most cases, is underdiagnosed in women. In contrast, in men, PVI is represented by varicocele, which is well-documented and diagnosed. PVI in women is not fully understood, but it is thought to be related to hormonal changes that occur during pregnancy, which can lead to increased blood flow and pressure in the pelvic veins [34]. The risk of developing PVI is also higher in women with conditions that increase intra-abdominal pressure, such as obesity, constipation, and chronic cough [35]. Varicose veins in the pelvic region are common, especially in women with multiple pregnancies; however, they are usually not diagnosed from the beginning, and from one pregnancy to the next, these veins are under continuous stress and, as a result, will become more prominent [36].

In this study, we observed that unilateral left ovarian vein embolization in the patient required low pain management and enabled effective post-interventional recovery. We established that the patient’s multiple pregnancies had no effect on pain management, but there is evidence that parity and increased pelvic blood flow during pregnancy are involved in the etiology of pelvic pain during and after pregnancy. At present, no study has demonstrated the effect of parity on endovascular embolization treatment success [20,21]. In summary, the diagnosis of PVI in women can be challenging, as there is no gold standard test for the condition. However, imaging studies such as ultrasound, computed tomography (CT), or magnetic resonance imaging (MRI) can be used to visualize the dilated veins in the pelvis. The treatment options for PVI include medical therapy with non-steroidal anti-inflammatory drugs (NSAIDs) and hormonal therapy, as well as minimally invasive procedures, such as embolization or sclerotherapy, to block or shrink the affected veins [37].

## 4. Conclusions

Female gonadal venous insufficiency is a rare pathology, often manifesting with pain in the lower abdomen, a clinical appearance that, in rare cases, can suggest an acute abdomen. Since the objective clinical signs are poor, misdiagnosis can often be encountered. A thorough anamnesis correlated with the clinical examination and imaging investigations are essential for a positive diagnosis. Once the diagnosis is established, the minimally invasive therapeutic approach is preferred, with interventional radiology procedures being the method of choice. An overall evaluation of the venous system to detect venous insufficiency with other topography and a periodic follow-up are essential for the favorable evolution of the case.

## Data Availability

The data generated in this study may be requested from the corresponding author.
